# Modulation of Pluripotency in the Porcine Embryo and iPS Cells

**DOI:** 10.1371/journal.pone.0049079

**Published:** 2012-11-08

**Authors:** Aida Rodríguez, Cinzia Allegrucci, Ramiro Alberio

**Affiliations:** 1 School of Biosciences, University of Nottingham, Loughborough, United Kingdom; 2 School of Veterinary Medicine and Sciences, University of Nottingham, Loughborough, United Kingdom; Michigan State University, United States of America

## Abstract

The establishment of the pluripotent ICM during early mammalian development is characterized by the differential expression of the transcription factors NANOG and GATA4/6, indicative of the epiblast and hypoblast, respectively. Differences in the mechanisms regulating the segregation of these lineages have been reported in many species, however little is known about this process in the porcine embryo. The aim of this study was to investigate the signalling pathways participating in the formation of the porcine ICM, and to establish whether their modulation can be used to increase the developmental potential of pluripotent cells. We show that blocking MEK signalling enhances the proportion of NANOG expressing cells in the ICM, but does not prevent the segregation of GATA-4 cells. Interestingly, inhibition of FGF signalling does not alter the segregation of NANOG and GATA-4 cells, but affects the number of ICM cells. This indicates that FGF signalling participates in the formation of the founders of the ICM. Inhibition of MEK signalling combined with GSK3β inhibition and LIF supplementation was used to modulate pluripotency in porcine iPS (piPS) cells. We demonstrate that under these stringent culture conditions piPS cells acquire features of naive pluripotency, characterized by the expression of *STELLA* and *REX1*, and increased in vitro germline differentiation capacity. We propose that small molecule inhibitors can be used to increase the homogeneity of induced pluripotent stem cell cultures. These improved culture conditions will pave the way for the generation of germline competent stem cells in this species.

## Introduction

Embryonic stem cells (ESC) can be captured in vitro in two distinct states of pluripotency known as “naive” and “primed” [1]. A distinguishing feature of naive cells is their capacity to integrate into a blastocyst and to contribute efficiently to germline chimeras. Importantly, naive stem cells can be established from mouse strains of non-permissive genetic background and can be modified genetically by homologous recombination [2]. ESC from non-rodent mammals share features of primed pluripotency, characterized by their FGF and Activin/Nodal dependence, and reduced capacity to contribute to chimeras [3], [4], [5], [6], [7]. This has led to the suggestion that establishing naive pluripotency may be beneficial for generating highly competent stem cells amenable to genetic modification. The conversion of primed cells to naive state by switching culture conditions was first reported in the mouse following the dissociation of epiblasts into single cells [8]. Established epiblast stem cell (EpiSC) lines, however, are much more resilient to this switch and cannot be converted to naive ESC simply by changing the culture conditions, unless additional factors are overexpressed [9], [10], [11]. Similarly, human ESC (hESC) cultured under stringent conditions to force the conversion to a naive state are highly unstable and cannot be cultured extensively [12], [13], [14]. These findings demonstrate that the naive state can be captured efficiently by including specific cytokines during the establishment of new cell lines from mouse blastocysts, or shortly thereafter by disrupting cell interactions [8]. However, once a cell exits the naive state and transits to a primed state, the reversion can only be restored artificially by specific factor overexpression. In non-rodent species naive ESC have not yet been isolated from pre-implantation embryos. Stem cell lines derived from primate [3], [15], rabbit [7], [16], and pig [4], [6] embryos share features of primed pluripotency, as described for mouse EpiSC [17], [18]. This seems to suggest that the naive state is either absent or very transient during embryo development in these species. A link between naive pluripotency and physiological diapause has been proposed as the underlying developmental mechanism that might explain why naive stem cells can be established from certain inbred mouse strains [19], but not from non-permissive mouse strains [20] or from other mammals [21]. Attempts to impose naive culture conditions failed in establishing stem cell lines in the pig, however when ICM cells from early blastocysts (day 5.5) were transduced with *KLF4*, cell lines with some features of naive pluripotency were established [22]. These lines failed to form teratomas when injected into SCID mice, but when *OCT-4* was over-expressed, the cells eventually acquired naive properties as demonstrated by their LIF dependency, teratoma formation capacity, and efficient integration to the ICM of blastocysts [22]. Thus, it seems that naive pluripotency can be imposed in cells derived from porcine embryos, however the optimal conditions for converting to this state may require species-specific considerations. For instance, NANOG protein is not detected in early pig blastocysts [23], [24], suggesting that entering the naive state in these cells might be compromised due to the lack of a functional pluripotency network.

Studies in mouse embryos show that modulation of MEK and Wnt signalling result in an enriched NANOG cell population in blastocysts [25], [26]. Interestingly, these effects were not observed in human and cattle embryos, where hypoblast cells expressing GATA4/6 were still detected [27], [28]. These interventions before the segregation of the inner cell mass (ICM) from trophectoderm (TE) offer an opportunity for capturing naive cells that may naturally only be present very transiently, if at all, when the ICM arises. The aims of the present study were 1- to study whether modulation of multiple signalling pathways can alter the proportion of NANOG positive cells in the ICM of pig blastocysts, and 2- to determine whether stringent culture conditions that support naive pluripotency in the mouse can be imposed in pig pluripotent cells.

## Materials and Methods

### Embryo Collection and In Vitro Culture

All the procedures involving animals have been approved by the School of Biosciences Ethics Review Committee (University of Nottingham, UK). Landrace × Large white crossbred sows were artificially inseminated twice over 2 days. Pig embryos were collected at day 4 after insemination. The oviduct and uterine horns were flushed with pre-warmed phosphate-buffered saline (PBS) supplemented with 1% fetal calf serum (FCS). The embryos were placed in an ovum concentrator and rinsed with PBS containing 1% FCS and 25 mM Hepes. Recovered embryos were allocated to either PZM3 [29] or N2B27 [28] culture medium supplemented with 0.3% fatty acid free BSA. Embryos at morula stage were included in the study. Embryos at earlier stages were cultured in PZM3BSA until the compact morula stage and subsequently transferred to the experimental groups. Embryos were incubated in a humidified atmosphere at 39°C, under 5%CO_2_ and 5%O_2._ The embryos were treated with inhibitors and growth factors at the following concentrations: PD0325901 (PD, MEK inhibitor, Calbiochem) 0.4 µM or 1 µM when combined with GSK3β inhibitor CHIR (GSK3β inhibitor, Selleck) 3 µM; PD173074 and PD161570 (FGF receptor inhibitors, Tocris) 100 nM; SB431542 (Type 1 TGFβ receptor ALK5, Tocris) 20 µM; 42009 (JAKi, JAK/STAT3 Inhibitor 420099, Calbiochem) 0.6 µM; LY294002 (InSolution™ LY 294002, Merck) 10 µM, human recombinant FGF4 (Peprotech) 1 µg/mL and heparin 1 µg/mL, as described by [27]. Heparin was included because it has been shown to bind FGF4, increasing the stability of the ligand-receptor interactions [30]. DMSO was used to dissolve the inhibitors, and was maintained at equal concentrations among groups. Control groups were added DMSO accordingly.

### Porcine Fetal Fibroblasts Isolation, Reprogramming and Cell Culture

Primary porcine fetal fibroblasts (PFF) cell lines were isolated from approximately 40 day-old fetuses. PFF were cultured in DMEM containing 10% fetal calf serum (FCS) and supplemented with 1% glutamine, 1% penicillin/streptomycin, 1% nonessential amino acids and 0.1 mM β-mercaptoethanol (culture medium: CM). Induced pluripotent stem (iPS) cells were generated from passage 3 cells. PFF were plated onto gelatinized dishes (0.1% porcine skin gelatine) at a density of 0.1 million cells per 9.6 cm^2^. PFF were infected with virus containing-medium twice at 48 h intervals. Lentiviruses were produced by transfecting HEK293 cells with doxycycline inducible FUW-tetO vectors (Addgene), encoding the human cDNA sequence of the four transcription factors (4 factors) OCT4, KLF4, SOX2 and C-MYC [31] plus FUW-M2rtTA (Addgene). The virus containing-medium was collected 48 h after transfection. The media containing the 4 factors and FUW-M2rtTA virus were pooled in equal volume, filtered through a 0.45 µm filter and supplemented with 4 µg/mL polybrene (Sigma-Aldrich).

PFF were passaged 48 h after the second transduction and cultured in CM supplemented with 10^3^ units/mL mouse LIF (ESGRO, Chemicon International). Cells were supplemented with 2 ug/mL doxycycline (Dox, Sigma-Aldrich). Between 1 and 4 weeks after Dox induction the colonies were picked and plated onto mouse embryonic fibroblast feeders. After two manual passages, piPS cells were dissociated with 1 mg/mL collagenase IV and passaged every 3–4 days at a ratio of 1∶5 or 1∶6. Pig iPS cells required continuous Dox supplementation for survival. A normal karyotype was determined for the cell lines used in this study (2*n* = 38). The FCS + LIF derived piPS were transferred to serum free N2B27 medium supplemented with 0.3% BSA, 10^3^ units/mL mouse LIF (ESGRO, Chemicon International), 1 µM PD0325901, 3 µM CHIR and 100 nM PD173074, as described previously [9].

### Immunocytochemistry and Alkaline Phosphatase Activity

Before fixation, expanded embryos were treated with 0.5% pronase for 1–2 min to remove the zona pellucida (ZP). Hatched embryos were washed in 1% PBS/BSA and fixed in 2.5% paraformaldehyde (PFA) for 15 min at room temperature (RT). After three washes in 0.2% Tween 1% PBS/BSA (PBST), embryos were permeabilized in 0.2% Triton X-100 PBS for 20 min at RT and blocked for 1 h at RT in 7% BSA and 5% donkey serum in PBST. Pig iPS cells were fixed in 4% PFA and subsequently permeabilized with 0.1% Triton X-100 in PBS and blocked in 5% PBS/BSA. Embryos and piPS cells were incubated overnight at 4°C with the following primary antibodies: NANOG (1∶400; rabbit polyclonal, Peprotech 500-P236), and GATA-4 (1∶200; goat polyclonal, Santa Cruz Biotechnology SC1237), OCT-4 (1∶100; goat polyclonal, Santa Cruz Biotechnology SC8628), STELLA (1∶60; rabbit polyclonal, Abcam ab19878), SSEA-1 (1∶50; Hybridoma bank, Iowa City), all in 5% PBST/BSA. The following antibodies were used in differentiated piPS cells: NESTIN (1∶100, rabbit polyclonal, Abcam), SOX17 (1∶100, goat polyclonal, R&D Systems), CYTOKERATIN-14 (1∶100, mouse monoclonal, Hybridoma bank, Iowa City), βIII-TUBULIN (1∶100, mouse monoclonal, R&D Systems), VIMENTIN (1∶100, AMF-17b, mouse monoclonal, Hybridoma bank, Iowa City). Pig iPS cells incubated with SSEA-1 antibody were not permeabilized. After 4 washes in 0.05% PBST-Triton (PBSTT) embryos and piPS cells were transferred to the appropriate secondary antibody and incubated for 1 h at RT, followed by 3× washes in PBSTT. Embryos and cells were mounted in Vectashield with DAPI (4′-6-diamidino-2-phenylindole; Vector Laboratories). Alkaline phosphatase (AP) activity was analyzed with the AP kit (Sigma-Aldrich) following the manufacturer’s instructions.

### Quantification of Total and ICM Cell Numbers in Embryos

The total cell number was obtained by counting all DAPI positive nuclei. Cell counts were performed in triplicates to obtain the average ± SD per embryo. The ICM cell number was calculated as the total number of NANOG and GATA-4 positive nuclei. Fluorescent images were acquired using epifluorescence.

### RNA Isolation and Polymerase Chain Reaction

RNA isolation was carried out using RNeasy kit (Qiagen) following the manufacturer’s instructions. RNA reverse transcription was performed using Omniscript synthesis kit (Qiagen). End-point PCR was performed with ReadyMix (Sigma-Aldrich) and 0.4 uM of each primer. PCR products were run in 1.5% agarose gel to determine the amplicon size. Quantitative RT-PCR (qRT-PCR) was performed using SYBR green mix (Roche) and 0.25 nM of each primer. For each gene, the analysis was performed in triplicate.

RT-PCR protocol included an initial step of 95°C (5 min), followed by 45 cycles of 10 sec at 95°C, 15 sec at 60°C and a primer extension step of 20 sec at 72°C. Fluorescence data were acquired at 72°C. Melting-curve analysis to confirm product specificity was performed immediately after amplification and the amplicon size was checked by electrophoresis. The relative expression of the target gene was normalized with *GAPDH* and a calibrator sample. Sequence accession numbers were obtained from NCBI, Ensembl and TGI databases. Primers used in this study are listed in Table 1.

**Table 1:** List of primers used in this study.

Gene	Primer sequence Fw		Amplicon	Accession number or	
	Primer sequence Rv		(bp)	[Reference]	
*β-ACTIN*		TCCCTGGAGAAGAGCTACGA	249		AJ312193
		CGCACTTCATGATCGAGTTG			
*GAPDH*		GGGCATGAACCATGAGAAGT	162		AF017079.1
		GTCTTCTGGGTGGCAGTGAT			
*OCT3/4*		GCAAACGATCAAGCAGTGA	201		NM_001113060
		GGTGACAGACACCGAGGGAA			
*NANOG*		TTCCTTCCTCCATGGATCTG	214		DQ447201
		ATCTGCTGGAGGCTGAGGTA			
*Sox-2*		AAGAGAACCCCAAGATGCACAACT	219		TC208722
		GCTTGGCCTCGTCGATGAAC			
*KLF4*		CCATGGGCCAAACTACCCAC	154		EU669075.2
		TGGGGTCAACACCATTCCGT			
*REX1*		TTTCTGAGTACGTGCCAGGCAA	201		TC206552
		GAACGGAGAGACGCTTTCTCAGAG			
*STELLA*		CTGAGTAGGTTGAGCCCACA	281		AJ656181.1
		CCAAAAGAGGCAAAACCTGA			
*FGF5*		GGAGCAGAGCAGCTTTCAGT	170		ENSSSCG00000009253
		ACAATCCCCTGAGACACAGC			
*NODAL*		CGTCTCCAGATGGACCTGTT	222		AM072821
		CTGCTCTGGAGAGAGGTTGG			
*VASA*		TCTTCCTATGTTCCCATCTTTG	254		NM_001001910.1
		TTGTTTGAAAAACCTCTGTTTCC			
*SOX17*		CGCACGGAGTTTGAACAATA	167		TC248504
		CAGACGTCGGGGTAGTTACAG			
*CARDIAC ACTIN*		CAGGTATTGCTGATCGCATGCA	201		TC270296
		ATTTGCGGTGGACGATGGA			
*PAX6*		CAGCTTCACCATGGCAAATA	197		TC269600
		GGGAAATGAGTCCTGTGGAA			
*NESTIN*		TACCTGGAAGCGGAAGAGAA	201		TC295480
		CTGATCCAGGTCTGCCTTGT			
*GATA6*		CAGGAAACGAAAACCTAAGAGCAT	201		TC238300
		TTCTCGGGATTAGCGCTCTC			
*FUW-hOCT4*		CCCCTGTCTCTGTCACCACT	148		[30]
		CCACATAGCGTAAAAGGAGCA			
*FUW-hSOX2*		ACACTGCCCCTCTCACACAT	122		[30]
		CATAGCGTAAAAGGAGCAACA			
*FUW-hKLF4*		GACCACCTCGCCTTACACAT	137		[30]
		CCACATAGCGTAAAAGGAGCA			
*FUW-hCMYC*		CAGCTACGGAACTCTTGTGC	125		[30]
		CCACATAGCGTAAAAGGAGCA			

### In Vitro Differentiation

Embryoid bodies (EB) were prepared from piPS cells using the hanging drop method. Briefly, piPS cells were trypsinized before preparing hanging drops at a density of 3000 cells/EB. The differentiation medium consisted of DMEM supplemented with 1 ug/mL Dox (for the first 10 days, and was subsequently removed), 1% glutamine, 1% penicillin/streptomycin, 1% nonessential amino acids and 20% FCS. Aggregated EBs were transferred to non-adherent dish after 4 days. After 10 days, EBs were plated onto gelatine coated plates and cultured for up to 30 days. EBs were collected and frozen at −80°C until analysis.

### Statistical Analysis

To evaluate the statistical differences in relative numbers, probability (P) values were established using Two-sided Mann-Whitney U test. Two-tailed Student’s *t*-test was used to compare absolute values between groups. Differences between groups were considered significant when *P*<0.05. Pearson’s correlations coefficient was used to analyse results from qPCR.

## Results

### Culture of Porcine Embryos to the Late Blastocyst Stage

**Figure 1:**
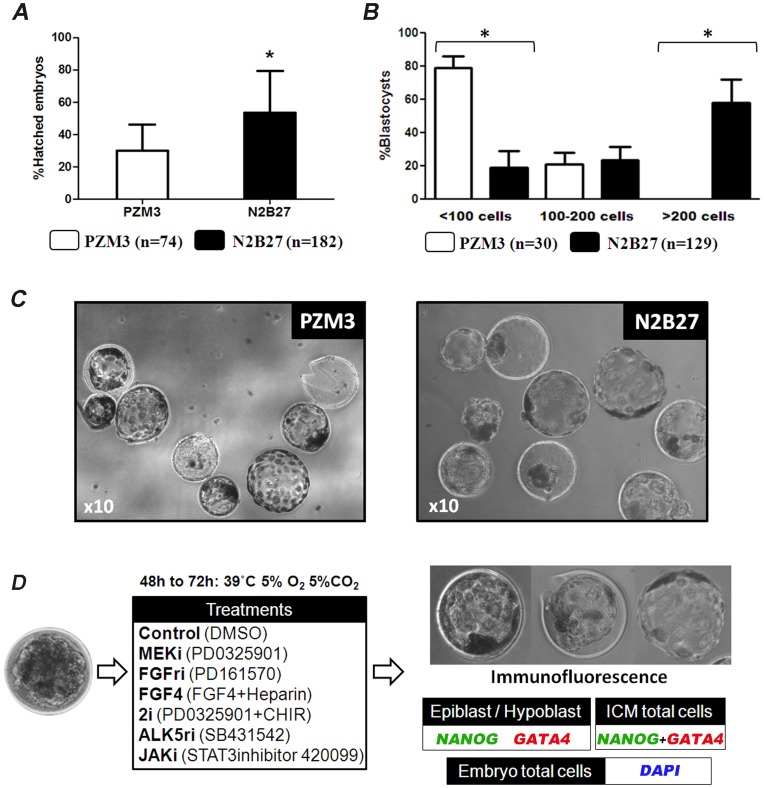
Development of pig morulae after in vitro culture. A) Proportion of embryos hatching from the zona pellucida after culture in PZM3 and N2B27. B) Cell count of blastocysts developing after culture in PZM3 and N2B27. C) Images of embryos obtained after incubation of morulae in PZM3 and N2B27. D) Diagram depicting the experimental design used to study signalling pathways involved in the segregation of ICM. * *P*<0.05.

To study the segregation of the ICM and TE, morulae obtained at day 4 of in vivo development were cultured in PZM3 medium under low oxygen, which has previously been shown to support in vitro porcine embryo development [29]. After 48 to 72 h, 30.2% (SD ±15.9, n = 74) of embryos hatched from the zona pellucida, and the majority of these (79% ±15.5) had a total cell number <100 cells (Fig. 1A,B). Because these embryos showed very small or no ICM (Fig. 1C), it was not possible to perform meaningful analysis of lineage segregation. Next we tested N2B27, a serum-free basal medium used for growing ESC and human embryos [28], [32]. After 48 to 72 h in culture, 53.4% (SD ±25.8, n = 182) of embryos hatched, and the total cell number exceeded 100 cells in more than 82% of these embryos (Fig. 1B). Since a clear ICM was visible in most embryos (Fig. 1C) this culture system was selected for the experiments performed in this study (Fig. 1D).

### Effect of FGF Signalling During Hypoblast Segregation in the Porcine Embryo

**Figure 2:**
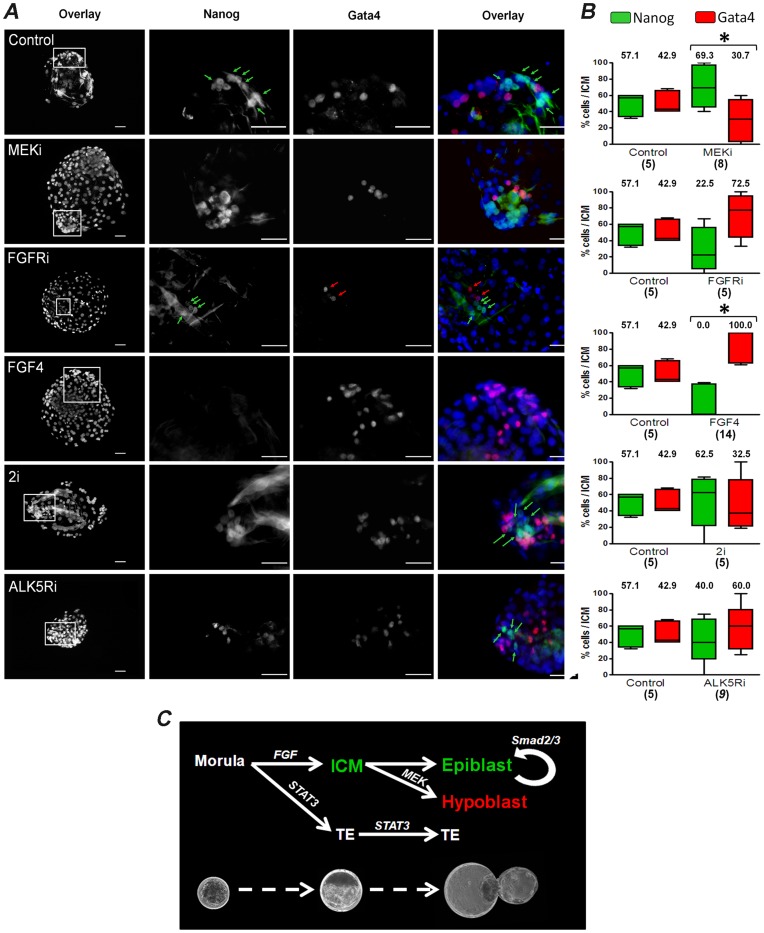
Effect of signalling inhibition in lineage segregation. A) Immunofluorescence images of NANOG and GATA-4 of hatched blastocysts after incubation with the indicated molecules from the morula stage. Blue: nuclei stained with DAPI. Scale bar: 50 µm. B) Box-whisker plots depicting the proportion of NANOG (green) and GATA-4 (red) cells of embryos treated with different molecules. Numbers indicate median values for each group. * *P*<0.05. C) Model depicting the stages of embryo development studied in this report. Inhibition of STAT3 signalling interferes with the cavitation process affecting the development of the ICM and the TE during the transition from early to late blastocyst. In addition, inhibition of FGF receptors at the morula stage prevents the formation of the ICM, whereas inhibition of MEK signalling reduces, but does not totally abolish, the segregation of hypoblast (GATA-4) in late blastocysts. Inhibition of SMAD2/3 does not interfere with the activation of NANOG in the blastocyst, but is needed for the development of the epiblast, as previously shown [4].

NANOG and GATA-4 are expressed in mutually exclusive cellular domains marking the epiblast and the hypoblast, respectively [25]. In the pig embryo these two factors are not found in early blastocysts (days 5.5–6.5) [23], [24], but they can be detected in the early epiblast from day 7.5 [33]. Since it is not clear whether NANOG and GATA-4 are expressed in mutually exclusive domains, in vivo retrieved morulae cultured for 48 h until they progressed to the late blastocyst stage were analysed by immunofluorescence (IF). Consistent with previous findings in mice and humans, NANOG positive cells were observed in clusters surrounded by GATA-4 positive cells (Fig. 2A control). Importantly, NANOG and GATA-4 cells were only detected in hatched embryos (late blastocysts) containing >100 cells (n = 5), all others with <100 cells (blastocysts and expanded blastocysts; n = 15) stained negative. In four expanded blastocysts we found NANOG positive cells (2, 2, 4, and 7 cells), but no GATA-4 cells (not shown), suggesting that GATA-4 cells may originate from a NANOG positive population in the ICM. These observations, combined with the finding that OCT-4 is expressed in the pig ICM [24], [34], show that the pluripotency network is established during the transition from early to late blastocyst in this species.

To study how this process is regulated, we evaluated this transition in embryos treated with small molecule signalling inhibitors. Experiments in mouse embryos show that MEK inhibition prevents hypoblast differentiation and enhances the number of Nanog expressing cells in the ICM, however in human and bovine embryos no specific inhibition of GATA4/6 was observed under these conditions [27], [28]. We found that a high proportion of pig morulae cultured with the MEK inhibitor PD0325901 progressed to the hatched blastocyst stage (75%; n = 20) (Table 2). These embryos had an average cell count of 197.2 (SD ±44.1, n = 14) cells and a similar proportion of TE cells as the control group, indicating that the viability of these embryos was not visibly compromised by MEK inhibition. NANOG and GATA-4 protein were detected in the ICM of treated embryos, however the proportion of GATA-4 cells was reduced compared to DMSO-only treated embryos (Fig. 2B). This result indicates that the hypoblast can segregate in the absence of MEK signalling, although the number of GATA-4 cells is reduced. In rodents, FGF initiates hypoblast differentiation via MEK signalling [35]. Therefore, to gain further insight into GATA-4 activation, embryos were cultured with the FGF receptor inhibitor (FGFRi) PD161570. Although the proportion of embryos progressing to the hatched blastocyst stage (55% ±21.2, n = 20) and the total cell count (215.8±42.7, n = 15) was not significantly different to those treated with PD0325901, only few embryos (5/15) formed an ICM, all of which had very few cells (Table 2). To confirm that this effect was not due to the specific inhibitor, we repeated experiments with another FGFRi PD173074, and similar results were obtained (data not shown). After immunostaining, we observed no differences in the proportion of NANOG and GATA-4 cells compared to the control group (Fig. 2B). This result indicates that FGF inhibition does not interfere with hypoblast segregation, but might interfere with the formation of the original founder population of the ICM. Since FGF can signal through phosphatidylinositol 3-kinase (PI3K), we evaluated whether inhibition of this pathway affected the founder cell population of the ICM. Embryos treated with PD0325901+ LY294002 hatched at a similar rate to control embryos (41.67%) and showed no differences in NANOG and GATA-4 staining (not shown).

**Table 2:** Developmental capacity of pig morulae incubated with small molecule inhibitors.

	Development rates			Total HB			HB with ICM	
	Morulae(N)	B (%)	HB (%)	N	Total cells	TE (%)	% (N)	ICM (%)
**Control**	18	90.3±1.9^a^	51.2±15.9^a^	10	111.3±25.9^a^	91.5±9.6	50.0 (5/10)	17.1±5.0^a^
**MEKi**	20	85.0±7.1^a^	75.0±7.1^a^	14	197.2±44.1^c^	93.3±6.0	57.1 (8/14)	11.1±3.7^bd^
**FGFRi**	26	79.4±29.1^a^	55.0±21.2^a^	15	215.8±42.7^c^	99.3±1.0	33.3 (5/15)	2.0±0.7^bc^
**FGF4**	20	94.4±7.8^a^	62.5±24.7^a^	15	209.7±78.2^c^	93.6±5.3	93.3 (14/15)	6.9±5.2^bd^
**2i**	20	96.9±4.4^a^	80.0±28.3^a^	15	220.8±73.8^c^	98.0±3.3	33.3 (5/15)	6.1±2.7^bc^
**ALK5Ri**	18	78.6±14.5^a^	65.5±17.3^a^	11	144.0±21.1^b^	89.6±9.3	81.8 (9/11)	12.7±8.7^ad^
**JAKi**	14	47.7±11.0^b^	7.1±10.1^b^	2	85.0±50.9^a^	98.3±2.3	50.0 (1/2)	3.3

a vs. b, P<0.05 compared to Control group.

c vs. d, P<0.05 compared to FGFRi group.

We next evaluated the effect of FGF4 supplementation in lineage segregation. Morulae incubated with FGF4 and heparin developed normally to the hatched blastocyst stage (62.5±24.7, n = 20) and had an average total cell number of 209.7 (SD ±78.2, n = 15; Table 2). The majority of these embryos formed a clear ICM, although it was significantly smaller compared to the control group. Furthermore, most embryos (9/14, 64.2%) had 100% GATA-4 positive cells, and no NANOG positive cells (Fig. 2A, B). This result shows that FGF4 promotes the segregation of the hypoblast without affecting the total cell number of the embryo. These experiments demonstrate that MEK inhibition has only a partial effect in preventing hypoblast segregation in the pig embryo (Fig. 2C).

### Effect of GSK3β, JAK/STAT3 and ALK5 Signalling Inhibition During ICM Segregation in the Porcine Embryo

We next evaluated whether a combination of MEKi (PD0325901) and stimulation of Wnt signalling by inhibition of GSK3β could contribute to an enrichment of the NANOG population in the ICM, as described in the mouse [25]. A moderate increase in the proportion of hatched embryos (80% ±28.3, n = 20) and in the total cell number (220.8±73.8, n = 15) was observed in these embryos (Table 2). The percentage of ICM/total cells was lower than in control embryos. The proportion of NANOG positive cells showed a median value similar to embryos treated with MEKi, however, due to the high variability between embryos, the observed differences were not statistically significant compared with the control (Fig. 2B). Thus, stimulation of Wnt signal together with MEK inhibition does not have a synergistic effect on the number of NANOG positive cells present in the porcine ICM.

LIF signalling is operative in the mouse ICM, as demonstrated by the requirement of this cytokine for the derivation of ESC. In the pig there is limited information on the role of this cytokine during the formation of the ICM, although recent studies show that LIF receptors are expressed in the TE of late blastocysts, but not in the ICM [23]. We found that hatching rate (7.1% ±10.1) was significantly reduced in embryos treated with JAK/STAT3 inhibitor 420099 compared to the control group, mainly because the embryos degenerated between early to late blastocyst transition (Table 2). Of the two blastocysts that developed, only one had an ICM with two NANOG positive cells. This result indicates that during the transition from morula to blastocyst JAK/STAT3 signalling plays a critical role in the development of the ICM and the TE.

Activin/Nodal signalling controls NANOG expression and is essential for maintaining hESC and mEpiSC in the undifferentiated state [4], [17], [36]. Furthermore, Activin A is expressed in the porcine ICM [37], suggesting that this pathway may participate in the activation of NANOG. To test this possibility we incubated morulae with the ALK5 receptor inhibitor SB431542, which causes downregulation of Nanog and neurectoderm differentiation in EpiSC [4], [17], [18]. We found that progression to the hatched blastocyst stage (65.5% ±17.3, n = 18) and total cells numbers were not affected by the treatment (Table 2). Similarly, the proportion of ICM cells and distribution of NANOG and GATA-4 cells was unaffected compared to the control group (Fig. 2A,B; Table 2). This result demonstrates that Activin/Nodal signalling is not required for NANOG activation and for normal segregation of the hypoblast.

### MEK and GSK3β Inhibition + LIF Promotes Naive Pluripotency in Porcine iPS Cells

The results above suggest that during the transition from morula to blastocyst the ICM responds to MEK inhibition by reducing the proportion of cells differentiating to hypoblast. The inhibition of this signalling pathway, together with inhibition of GSK3β (known as 2i medium) and activation of JAK/STAT3 pathways, are essential for establishing naive pluripotency in the mouse [10], [32]. Here we applied these culture conditions to embryos treated with 2i between the morula to blastocyst transition. Seven whole-embryos plated onto feeders in 2i + LIF medium attached after 24 h, however in the following 72 h most clumps failed to initiate proliferation and degenerated (not shown). This result suggests that the conditions for the isolation of naive cells directly from embryos are still sub-optimal to support stem cell derivations.

**Figure 3:**
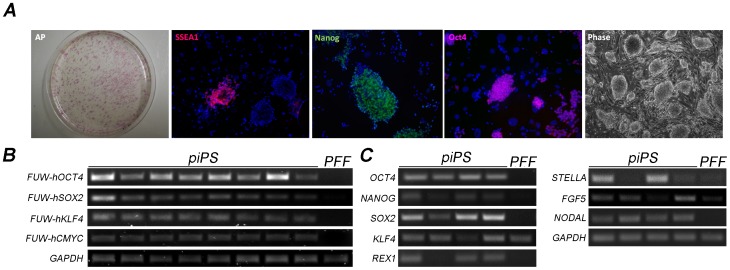
Characterization of piPS cells. A) Alkaline phosphatase, SSEA1, NANOG and OCT-4 staining in colonies of piPS cells. Phase contrast image shows the morphology of cells under low magnification. B) Expression of exogenous factors was determined by RT-PCR in 8 cell lines. PFF: porcine fetal fibroblasts used for generating the iPS cells. C) RT-PCR analysis of four iPS cell lines shows variables levels of gene expression of endogenous genes.

We turned to a piPS cell system to study whether the naive state could be imposed in pluripotent cells in this species. A recent report shows that piPS cells become LIF dependent when cultured in normal ESC culture conditions [38]. Here we extended these investigations to determine whether 2i (or 3i) + LIF could be used to force naive features in piPS cells. We generated iPS cells from porcine fetal fibroblasts using *hOCT-4, hc-MYC, hKLF4* and *hSOX2* under the control of doxycycline [31]. Colonies started to appear after 6–7 days, and after 15–30 days strong alkaline phosphatase activity was detected in most cell clumps (Fig. 3A). Colonies were picked and transferred onto feeder cells in standard ESC culture medium (containing 10% FCS and LIF). Eight cell lines were expanded and further characterized. Cells under these conditions grew as compact colonies which consistently expressed OCT-4 and endogenous NANOG, but only few colonies expressed SSEA1 antigen (Fig. 3A). All the lines expressed the four exogenous factors (Fig. 3B), therefore we selected four lines to characterize their gene expression profile in more detail. A distinguishing feature of mouse naive stem cells is the expression of *Stella* and *Rex1*, in contrast to the expression of *Fgf5* and *Nodal*, typical of primed stem cells [8], [17]. An initial analysis of these lines showed that all lines expressed endogenous *OCT-4, NANOG, SOX2, KLF4, NODAL and FGF5,* however *STELLA* and *REX1* were expressed in 2/4 lines (Fig. 3C). These results show that piPS cells cultured with FCS and LIF are heterogeneous, displaying features of primed and naive pluripotency.

**Figure 4:**
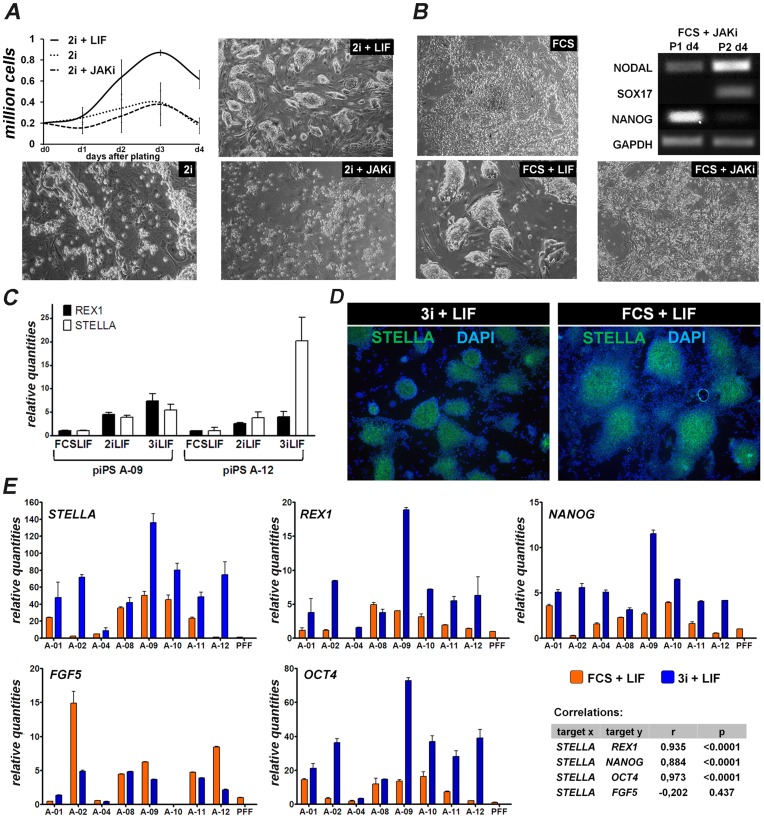
Induction of naive features in piPS cells cultured with 2/3i + LIF. A) piPS cells were seeded in the indicated culture conditions and their growth was quantified daily for 4 days. B) piPS cells were seeded in the indicated culture conditions and collected 4 days after seeding at passage 1 and 2. RT-PCR was performed to analyse gene expression. C) qRT-PCR for *REX1* and *STELLA* of two cell lines grown in FCS + LIF or in 2/3i + LIF. Expression was normalized to FCS + LIF piPS cells. D) STELLA immunostaining in piPS colonies seeded in the indicated culture conditions. E) qRT-PCR for *STELLA*, *OCT-4*, *NANOG*, *REX1* and *FGF5* of 8 iPS cell lines grown in FCS + LIF or in 3i + LIF. Expression was normalized to PFF sample. Pearson’s correlation coefficient analysis was performed to study interactions between genes.

We next tested whether switching the culture conditions of piPS cells to 2i and 3i (2i + FGFRi) + LIF promoted features of naive pluripotency, as previously demonstrated for mouse ESC [32]. Switching piPS cell culture medium to N2B27 supplemented with 2i (or 3i) and LIF resulted in some cell death during initial 1–2 passages, however in subsequent passages the cells grew as homogeneous round colonies (Fig. 4A). To test their LIF requirements cells were cultured either with 2i only or with the JAK/STAT3 inhibitor. In both conditions extensive cell death was observed and most cells were lost within one passage (Fig. 4A). Interestingly, LIF withdrawal or JAK/STAT3 inhibition of cells grown with FCS resulted in a rapid loss of their compact morphology. These cells showed overt signs of differentiation, which was confirmed by the expression of *SOX17* and *NODAL* and the reduction in *NANOG* expression (Fig. 4B). Similar results were obtained with cells cultured with 3i (not shown). These experiments demonstrate that piPS cells depend on LIF to maintain the undifferentiated state.

We next evaluated whether cells cultured in 2i/3i + LIF activated genes indicative of naive pluripotency. Two cell lines (A-09 and A-12) transferred from FCS + LIF to 2i/3i + LIF showed increased *STELLA* and *REX1* expression within 2 passages (Fig. 4C). Cells grown in 3i + LIF showed a similar phenotype to cells grown in 2i + LIF, but overall the cultures in 3i were more homogeneous and showed low levels of differentiation (not shown). Immunolocalization of STELLA demonstrated homogeneous staining in colonies of cells grown in 3i + LIF, whereas cells grown in FCS + LIF had a large proportion of cells in the periphery of the colonies that were STELLA negative (Fig. 4D). Analysis of additional 6 lines shows that 3i + LIF induced an increase in *STELLA* expression in all cell lines, however high variability was detected between different lines (Fig. 4E). Interestingly, the changes in *STELLA* expression correlated with changes in *NANOG, OCT-4* and *REX1* expression, but were not correlated with *FGF5* expression (Fig. 4E).

### Naive piPS have Increased Germline Differentiation Potential

**Figure 5:**
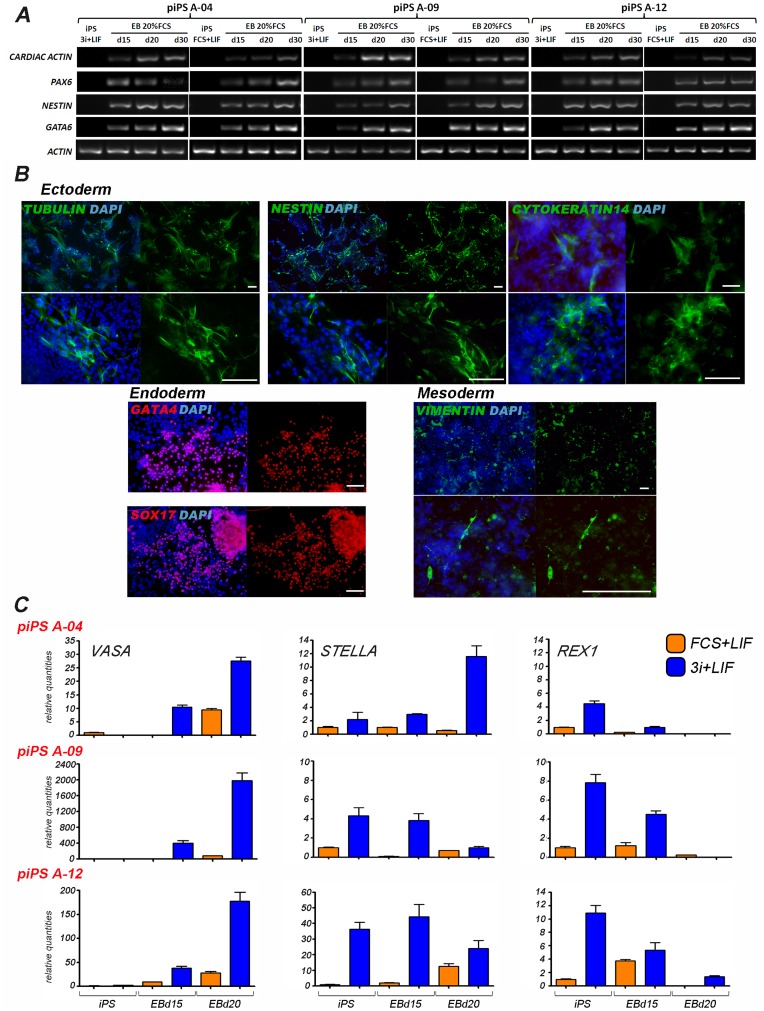
Differentiation potential of iPS cells grown under different conditions. A) RT-PCR performed on three iPS cell lines (A04, A-09, and A-12) grown under 3i + LIF or FCS + LIF were spontaneously differentiated in medium containing 20% FCS. Gene expression analysis was performed at the indicated time points. B) Immunocytochemistry analysis was carried out in spontaneously differentiated piPS cells. Scale bar: 10 µM. C) qRT-PCR analysis of three iPS cell lines induced to differentiate in culture medium containing 20% FCS over 20 days. Expression levels are relative to iPS cells grown in FCS + LIF.

Mouse naive stem cells can differentiate to all somatic lineages and can generate germline chimeras efficiently, in contrast to primed stem cells. We investigated the capacity of three piPS cell lines maintained in FCS + LIF or in 3i + LIF media to differentiate in EBs. Expression of ectoderm (*PAX6, NESTIN*), mesoderm (*CARDIAC ACTIN*), and endoderm markers (*GATA6*) was determined by RT-PCR in the three cell lines analysed (Fig. 5A). The expression of markers of ectoderm (β-TUBULIN, NESTIN, CYTOKERATIN 14), mesoderm (VIMENTIN), and endoderm (SOX17, GATA4) was further confirmed by immunohistochemistry (Fig. 5B).

We next compared the germ line differentiation capacity of cells grown in 3i + LIF vs. FCS + LIF. Three cell lines expressing *STELLA* at different levels (A-04*^low^* (female line), A-09*^medium^* (male line), and A-12*^high^* (female line)) when cultured in 3i + LIF (Fig. 4E), were induced to differentiate for 20 days. The differentiation capacity of these cells was compared to the respective cell lines grown in FCS + LIF before inducing differentiation. *VASA* was induced after 15 days of differentiation in the three cell lines cultured in 3i + LIF, however no *VASA* expression was detected when the same lines were grown with FCS + LIF (Fig. 5C). After 20 days, *VASA* expression increased further in cells grown in 3i + LIF, while some expression was also detected in cells grown in FCS + LIF at this timepoint. To determine whether the differentiation protocol induced the exit from the naive state, *REX1* was also analysed. Cells cultured in 3i + LIF had higher levels of *REX1* before differentiation, however a sharp reduction was detected after 15 days, and it was not detected in two (A-04 and A-09) of the three cell lines after 20 days. These results show that piPS cells expressing *STELLA* can be efficiently induced to initiate the germ cell differentiation program in vitro.

## Discussion

This study was designed to investigate the consequences of modulating signal transduction pathways in the establishment of pluripotency during pig embryo development and in piPS cells. The first set of experiments were designed to determine which signals participate in the establishment of NANOG expressing cells from in vivo produced embryos. Since NANOG protein is first detected in pig late blastocysts (from day 7.5) [33], the culture conditions were optimized to ensure a high proportion of embryos reaching this stage in vitro. The majority of embryos cultured in the conventional PZM3 culture medium did not progress beyond the blastocyst stage, had low total cell count, and very small or inexistent ICM. The culture of pig morulae in N2B27 resulted in a significant increase in hatching rate and total cell count, and supported cultures up to the late blastocyst stage in vitro. Furthermore, NANOG and GATA-4 cells were detected in the ICM of most hatched blastocysts. In earlier embryos (non-hatched) GATA-4 was never detected, however NANOG was found in a few isolated cells in 4/32 embryos, suggesting that NANOG expression precedes GATA-4 expression in the founder population of the porcine ICM. Since GATA-4 is a hypoblast marker, this observation also suggests that hypoblast cells (GATA-4 positive) segregate from early epiblast cells (NANOG positive).

We next investigated whether interfering with MEK signalling during morula-blastocyst transition prevented hypoblast segregation [25], [26]. In agreement with the observations in human and bovine embryos [27], [28], MEK inhibition did not completely prevent hypoblast formation in the porcine embryo. Although GATA-4 expression was still detected after MEK inhibition, the number of positive cells was significantly reduced. In contrast, a significantly higher number of NANOG positive cells were observed. Since the number of ICM cells was not affected by the treatment, the results demonstrate that the reduction in GATA-4 was due to a partial interference with hypoblast segregation. To establish whether MEK signal is triggered by a response to FGF, as previously shown in mouse embryos [25], [26], pig morulae were cultured with an FGFRi. Interestingly, inhibition of FGFR resulted in a significant reduction in the number of ICM cells, however the proportion of NANOG and GATA-4 cells was unaffected. These findings are in agreement with the observations in bovine embryos [27], although the effect of the treatment on total cell numbers was not reported in this study. Our results show that MEK-independent FGF signalling is important for the establishment of the founder population of the ICM. Since inhibition of MEK and PI3K signalling (an alternative downstream pathway of FGF) did not affect lineage segregation, it is possible that other FGF signalling effectors, such as phospholipase C and protein kinase C [39], may be playing a role in establishing the ICM. Future studies will focus on the role of these pathways on the formation of the ICM.

The role of FGF signalling in promoting hypoblast differentiation was further demonstrated in experiments where FGF4+ heparin were added to developing embryos. Treated embryos developed normally and maintained normal cell numbers in the ICM, however a significant increase in GATA-4 cells was observed. These results are in agreement with observations in mouse, rat, human and bovine embryos, and demonstrate that the role of FGF in promoting hypoblast segregation is conserved in mammals.

We next asked whether other signalling pathways may participate in the activation or maintenance of NANOG expression in the ICM. The role of Wnt was investigated first, since maintenance of this pathway in mouse ESC promotes cell proliferation and undifferentiated state when combined with a MEKi in serum free cultures [32]. In the pig embryo, inhibition of GSK3β in combination with a MEKi did not affect the segregation of hypoblast cells, indicating that this pathway is not critical during the establishment of the founder cells of the ICM.

The role of Activin/Nodal signalling was also evaluated, based on a previous study showing the transient expression of Activin A in porcine blastocysts [37] around the time when NANOG expression is activated. Inhibition of Activin/Nodal signalling did not affect the activation of NANOG, the number of ICM cells or the segregation of the hypoblast. This demonstrates that an Activin/Nodal-independent NANOG cell population exists during the formation of the porcine ICM, perhaps with features similar to the mouse ICM.

Since previous studies showed no increase in blastocyst rates of porcine and bovine embryos cultured with LIF [40], [41], here we tested the possible involvement of this signalling pathway through the inhibition of the JAK/STAT3 phosphorylation. We found that most embryos cultured in the presence of the JAK/STAT3 inhibitor failed to hatch from the zona pellucida and had significantly lower total cell count and ICM cells, indicating that blocking this signal directly interferes with the cavitation process. Indeed, a recent study described the expression of LIF receptors in the TE, but not in the ICM of porcine late blastocysts [23]. This suggests that LIF signalling may play an important role during the segregation of the TE, rather than with the formation of the ICM. It cannot be excluded that this effect is due to the interference with IL-6 (which signals through JAK/STAT3) that is produced in an autocrine manner, and plays a role during pig early embryo development [42].

These experiments demonstrate that the transition from morula to late blastocyst is characterized by the formation of the ICM, in which NANOG is first activated. The ICM then gradually expands before segregating GATA-4 cells. Although the activation of NANOG in the founder population appears to be independent of MEK, Wnt and Activin signalling, it is clear that FGF plays a crucial role in ensuring the expansion of the starting ICM population. These findings suggest that NANOG activation is a cell autonomous process, and that extrinsic signals (including FGF, TGFβ, Activin, and Wnt ligands) may cooperate during the transition from early to late epiblast to maintain NANOG activity and pluripotency. As defined in ESC, modulation of different signalling pathways supports two known states of embryonic pluripotency: naive and primed pluripotency [1]. A naive state, imposed by MEK and GSK3β inhibition + LIF cannot be used to capture NANOG-only ICM cells in porcine embryos, suggesting that additional signals may be needed to prevent hypoblast formation. Importantly, however, we show that naive state can be imposed effectively in piPS cells. These naive cells are LIF dependent, resembling the cells reported by others [22], [38], however the analysis of *STELLA* and *REX1* expression showed significant heterogeneity between piPS cell lines. *STELLA* and *REX1* expression are good markers of naive stem cells, and are not expressed in primed cells [8], [32], [43]. In agreement with findings in mouse cells, *STELLA* and *REX1* expression increased in piPS cells transferred to 2/3i + LIF conditions. From our results we conclude that *STELLA* expression is indicative of higher homogeneity in iPS cell cultures, which is reflected in the compactness of the colonies, low numbers of STELLA negative cells, and increased expression of *NANOG, OCT-4,* and *REX1*. A test comparing the differentiation capacity of *STELLA^high^*(cells in 3i + LIF) versus *STELLA^low^* (cells in FCS + LIF) cells demonstrated that both lines could differentiate to the three somatic lineages, but importantly, *STELLA^high^* cells had increased capacity to differentiate to *VASA*-expressing germ cell precursors. These findings lead us to suggest that naive pluripotency in piPS cells can be imposed in 2i and 3i + LIF conditions, conferring these cells increased in vitro differentiation capacity to germ line precursors. Future in vivo studies will determine the capacity of these cells to colonize chimeric embryos.
